# Modulation of Biointeractions by Electrically Switchable Oligopeptide Surfaces: Structural Requirements and Mechanism

**DOI:** 10.1002/admi.201300085

**Published:** 2014-01-25

**Authors:** Chun L Yeung, Xingyong Wang, Minhaj Lashkor, Eleonora Cantini, Frankie J Rawson, Parvez Iqbal, Jon A Preece, Jing Ma, Paula M Mendes

**Affiliations:** School of Chemical Engineering, University of BirminghamEdgbaston, Birmingham, B15 2TT, UK E-mail: p.m.mendes@bham.ac.uk; School of Chemistry and Chemical Engineering, Nanjing UniversityNanjing, 210093, P. R. China E-mail: majing@nju.edu.cn; School of Chemistry, University of BirminghamEdgbaston, Birmingham, B15 2TT, UK; Laboratory of Biophysics and Surface Analysis, School of Pharmacy, University of NottinghamUniversity Park, Nottingham, NG7 2RD, UK

**Keywords:** switchable surfaces, self-assembled monolayers, surface plasmon resonance, electrochemistry, molecular dynamic simulations

## Abstract

Understanding the dynamic behavior of switchable surfaces is of paramount importance for the development of controllable and tailor-made surface materials. Herein, electrically switchable mixed self-assembled monolayers based on oligopeptides have been investigated in order to elucidate their conformational mechanism and structural requirements for the regulation of biomolecular interactions between proteins and ligands appended to the end of surface tethered oligopeptides. The interaction of the neutravidin protein to a surface appended biotin ligand was chosen as a model system. All the considerable experimental data, taken together with detailed computational work, support a switching mechanism in which biomolecular interactions are controlled by conformational changes between fully *extended* (“ON” state) and *collapsed* (“OFF” state) oligopeptide conformer structures. In the fully *extended* conformation, the biotin appended to the oligopeptide is largely free from steric factors allowing it to efficiently bind to the neutravidin from solution. While under a *collapsed* conformation, the ligand presented at the surface is partially embedded in the second component of the mixed SAM, and thus sterically shielded and inaccessible for neutravidin binding. Steric hindrances aroused from the neighboring surface-confined oligopeptide chains exert a great influence over the conformational behaviour of the oligopeptides, and as a consequence, over the switching efficiency. Our results also highlight the role of oligopeptide length in controlling binding switching efficiency. This study lays the foundation for designing and constructing dynamic surface materials with novel biological functions and capabilities, enabling their utilization in a wide variety of biological and medical applications.

## 1. Introduction

Materials and surfaces with stimuli-responsive properties, which mimic biology,^1^ are being developed experimentally for biomedical applications.^2^ Active and switchable surfaces comprising domains that confer tailored responsiveness are playing an important part in the development of tissue engineering scaffolds,^3^ highly sensitive biosensors,^4^–^6^ novel drug delivery systems,^7^,^8^ and highly functional microfluidic, bioanalysis, and bioseparation systems.^9^–^12^

Physical stimuli, such as temperature,^13^,^14^ light,^15^,^16^ magnetic field^17^ and electrical potential,^18^–^20^ are able to alter and manipulate the surface properties and, thus, change and control function and activity of biomolecules on surfaces. Switchable self-assembled monolayers (SAMs) that can control biomolecular interactions using an electrical stimulus are particularly appealing because of their fast response times, ease of creating multiple individually addressable switchable regions on the same surface and low-driven voltage or electric field that are compatible with biological systems.^21^ Electrically switchable SAMs have been demonstrated to display controllable switching properties that can modulate the interactions of surfaces with proteins,^18^–^20^ DNA,^22^,^23^ and mammalian^21^ and bacterial^24^ cells.

Particularly interesting are the electrically switchable oligopeptide mixed SAMs, which enable controlled protein interactions with surfaces.^20^ These switchable mixed SAMs are composed of a two molecular components, (i) a positively charged 4-mer of lysine (K) that is functionalized at one end with biotin, which recognises the neutravidin protein, and at the other end with a cysteine (C), for binding to gold substrates (biotin-4KC), and (ii) an ethylene glycol-terminated thiol (to space out the oligolysine peptides). The salient feature of the oligolysine peptide mixed SAMs is that they exhibit protonated amino side chains at pH 7, providing the basis for the switching “ON” and “OFF” of the biological activity on the surface by an electrical potential. These SAMs have been shown to regulate the binding between the biotin ligand on the surface and neutravidin from solution. Although these SAMs have been demonstrated to be capable of switching upon exposure to an electrical potential and so possess unique advantages in terms of addressability, the nature of the conformational changes and the dynamics that regulate biomolecule activity are unknown.

To design any reliable or predictable surface material based on such oligolysine molecular switch, it is essential to have a detailed understanding of the structural requirements and mechanism by which the oligopeptides on the surface alter the ligand function. This would allow the molecular architecture in a surface material to be optimized to maximise the performance and efficiency of the switching of biomolecular interactions.

**Scheme 1:**
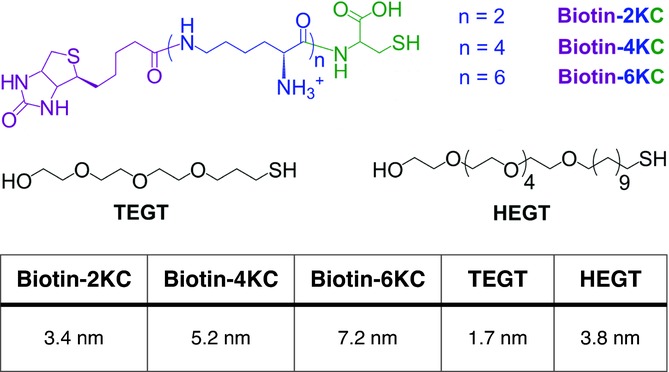
Chemical structures of the oligopeptides (biotin-2KC, biotin-4KC and biotin-6KC) and ethylene glycol-terminated thiols used in the mixed SAMs, and their molecular lengths in fully extended conformations.

Toward these goals, we report here detailed studies performed by X-ray photoelectron spectroscopy (XPS), surface plasmon resonance spectroscopy (SPR) and molecular dynamic simulations on the oligopeptide:ethylene glycol-terminated thiol mixed SAMs. While SPR offers a system to retrieve information such as binding ability and binding switching efficiency, atomic molecular dynamic simulations provide molecular insights into the electrical-induced conformational changes of the oligolysines within the SAM. One of the components of the SAM is the end functionalised biotinylated oligolysine peptide described above – biotin-4KC (**Scheme** **1**). A tri(ethylene glycol)-terminated thiol (TEGT) is used as the second SAM component.

Here we consider how the ratio of these two components can affect the binding switching efficiency. We follow with experiments and theoretical studies detailing the role that the length of the oligolysine has on the switching ability of the biotin ligand, whereby we incorporate onto the oligopeptide two less or two more lysine residues, relative to biotin-4KC, namely, biotin-2KC and biotin-6KC (Scheme 6).

## 2. Results and Discussion

In the search for an understanding of the relationship between oligopeptide composition in the mixed SAM and its switching ability, biotin-4KC:TEGT mixed SAMs on gold were prepared at different solution ratios ranging from 1:1 to 1:500 (**Table** **1**). X-ray photoelectron spectroscopy (XPS) confirmed the formation of the mixed SAMs, showing signals from C (1s), O (1s), S (2p) and N (1s) (Figure S1 in the Supporting Information). By integrating the area of the S (2p) and N (1s) peaks for the mixed monolayers (the biotin-4KC oligopeptide consists of 11 N atoms and 2 S atoms, whereas TEGT has no N and 1 S atom only), we were able to calculate the ratio of biotin-4KC to TEGT on the surface. The samples were found to be reproducible. As expected an increased amount of TEGT in the mixed solution has led to an increase of this component in the mixed SAM. Nevertheless, as reported previously for other mixed SAM systems,^25^–^27^ the composition of the mixed solution does not directly equal the composition of the mixed SAMs on the surface. Correlation analysis indicated a logarithm relationship between the biotin-4KC:TEGT solution and surface ratios, with the biotin-4KC being significantly enriched in the mixed SAM in comparison to its solution composition. This has been a general trend observed when thiols with different chain lengths have been used to form monolayers, in which the longer chain component is preferentially adsorbed, suggesting a predominantly thermodynamic control of the adsorption.^25^,^26^

**Table 1:** Biotin-4KC:TEGT solution ratios and respective surface ratios calculated using XPS. Binding capacity and switching efficiency as determined by SPR analysis

Biotin-4KC:TEGT ratio		Binding capacity (RU)	Switching efficiency (%)
		
Solution	Surface		
1:0	1:0	3553 ± 258	7 ± 2
1:1	1:3 ± 3	3192 ± 164	27 ± 3
1:10	1:5 ± 2	3053 ± 69	34 ± 5
1:40	1:16 ± 4	2195 ± 161	90 ± 3
1:100	1:22 ± 8	1492 ± 72	62 ± 8
1:500	1:38 ± 6	1375 ± 75	60 ± 4

**Figure 1:**
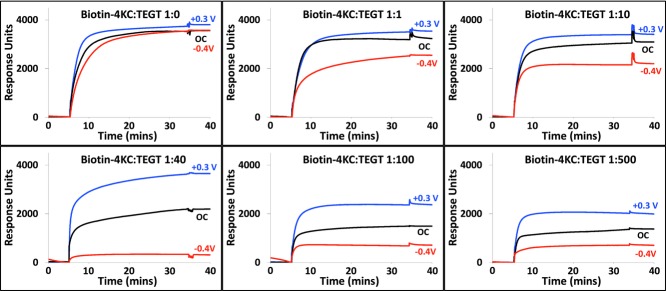
SPR sensorgram traces showing the binding of neutravidin (37 μg.mL^−1^) to the biotin-4KC:TEGT mixed SAMs at solution ratios of 1:0, 1:1, 1:10, 1:40, 1:100 and 1:500 under open circuit conditions (no applied potential), an applied positive (+0.3 V) and negative (−0.4 V) potential.

We next assessed the binding capacity and switching efficiency of the biotin-4KC:TEGT mixed SAMs, by analysing the binding events between the biotin ligand on the mixed SAM and neutravidin using SPR (**Figure** **1**). Neutravidin is a protein that consists of four identical subunits, each binding one biotin with extremely high affinity (K_a_ ≈ 10^13^ M^−1^).^28^ In the SPR experiments, the mixed SAMs were exposed to a flow of PBS, to establish the baseline, followed by an injection of neutravidin in PBS into the SPR flow cell for 30 min. The SPR flow cell was then flushed with PBS to leave only the specifically bound neutravidin on the biotinylated mixed SAM. The binding capacity is defined as the difference in the SPR response units between the beginning of injection of protein and the end of washing with PBS.

The biotinylated mixed SAMs exhibited a high protein binding capacity, which upon a reduction of the amount of biotinylated peptide on the surface decreased significantly. Nevertheless, the reduction in the binding capacity was not proportional to the decrease in the amount of biotinylated peptide on the surface, indicating that steric hindrance and limited mobility of the densely packed biotin molecules might limit the protein binding to the biotin sites at low ratios of TEGT to Biotin-4KC.^29^

It is important to note that the binding capacity is also dependent on the length of the ethylene glycol thiol. Experiments conducted on mixed SAMs comprising the Biotin-4KC and a longer ethylene glycol thiol— HEGT in Scheme 6—has led to a greatly reduced binding of neutravidin to the biotinylated surface. Biotin-4KC:HEGT solution ratios were varied between 1:10 and 1:100, leading to surface ratios between 1:9 ± 4 and 1:19 ± 4, respectively. The neutravidin binding amount was essentially independent of the surface ratio used, with SPR signals in the range of 275–325 response units for all the surfaces.

Taking into consideration that the lengths of the biotin-4KC and HEGT, in fully extended conformations, are 5.2 nm and 3.8 nm, respectively, to a certain extent the biotin functionalities are expected to protrude from a matrix of HEGTs even if most likely both molecules adapt a rather unstretched form on the surface. Nevertheless, and based on the above-mentioned SPR results, there is strong evidence that the biotin moieties are not accessible for binding. We propose therefore that the suppression of biorecognition with the biotin-binding pockets of neutravidin is a result of the biotin moieties not standing further away from the HEGT matrix, thus not allowing complete insertion of the biotin into the binding pockets.

This reasoning is in line with previous studies that showed that increasing the length of the biotin linker in a mixed SAM increased the protein binding efficiency.^30^ Our hypothesis is also consistent with X-ray crystallographic analysis that revealed that the biotin is buried quite deeply inside the neutravidin barrel,^31^ indicating that the binding of biotin by neutravidin requires the complete insertion of the ligand into the binding pocket of the protein. TEGT in a fully extended conformation exhibits a length of 1.7 nm, approximately three-fold shorter than the biotin-4KC, allowing complete insertion of the biotin into the binding pocket and efficient binding of the neutravidin to the biotinylated monolayer.

Switching efficiency studies on the Biotin-4KC:TEGT mixed SAMs were conducted by monitoring, using electrochemical SPR spectroscopy, neutravidin binding to the biotinylated SAM to which a positive or negative potential was applied (Figure 1). Previously, we have demonstrated that the bioactivity of oligopeptide mixed SAMs can be controlled by application of +0.3 V (bioactive “ON” state) or –0.4 V (bio-inactive “OFF” state), while not affecting the SAM integrity. Thus, similar electrical potentials were used in these studies. While applying +0.3 V or –0.4 V, the baseline for the SAM gold chip was established using PBS, following which the neutravidin was introduced. Data were collected for 30 min, after which the surface was rinsed with PBS. The switching efficiency was calculated by dividing the difference in binding capacity between +0.3 V and –0.4 V by the binding capacity at +0.3 V.

**Figure 2:**
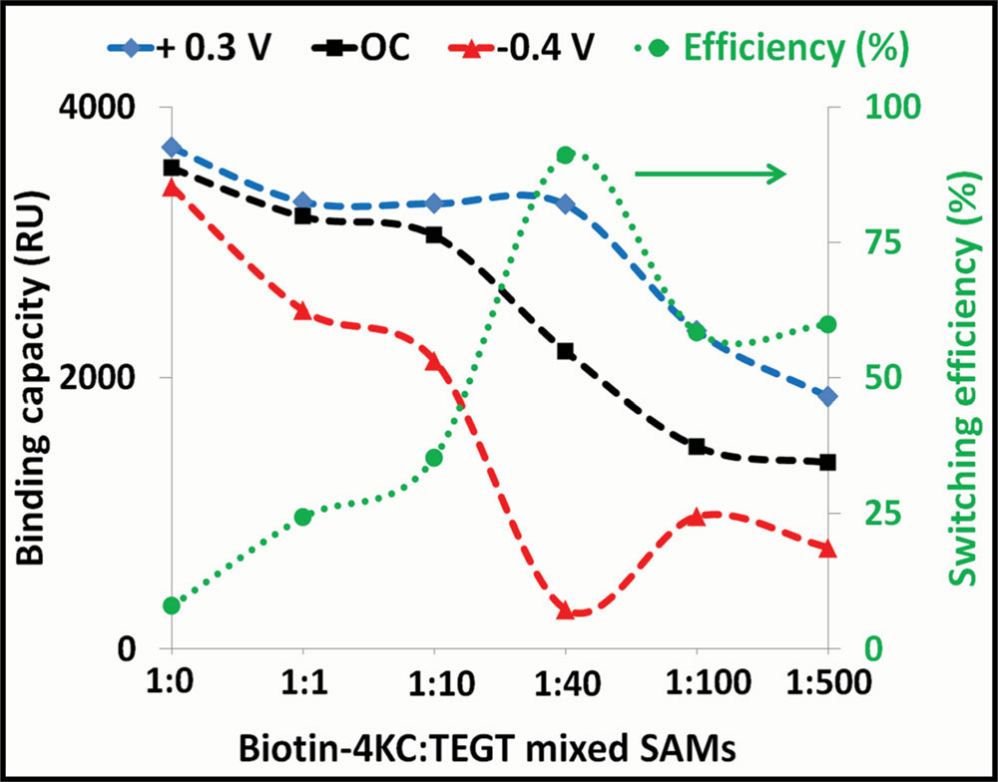
The binding capacity under open circuit conditions, an applied positive (+0.3 V) and negative (−0.4 V) potential as well as switching efficiency of the biotin-4KC:TEGT mixed SAMs at solution ratios of 1:0, 1:1, 1:10, 1:40, 1:100 and 1:500.

To begin with, we investigated the binding efficiency of the pure biotin-4KC monolayers (Figure 1). There were, however, no significant changes in response units observed in both +0.3 V and –0.4 V compared to that of the OC conditions, indicating that regulation of biomolecular interactions is not possible with a very high density of biotinylated oligopeptides on the surface. At progressively lower densities of the oligopeptide, the application of +0.3 V or –0.4 V started to result in some control over the binding of the protein to the biotin ligand presented at the surface. Nevertheless, the binding switching efficiency was shown to be limited at higher biotin-4KC:TEGT solution ratios, i.e. <1:10 (Table1 and **Figure** **2**). These results illustrated that the oligopeptide should be presented at optimum ratio on the surface such that binding capacity and switching efficiency can be maximised. For the biotin-4KC:TEGT mixed monolayers, the optimum surface ratio was identified as being in the order of 1:16, i.e. a mixed ratio from solution of 1:40 as depicted in Figure 2.

To rationalize the binding and switching properties of the biotinylated mixed SAMs at different ratios, we propose that the activation/inactivation switching mechanism is related to conformational changes of the oligopeptide on the surface induced by an electrical potential, involving first among all reorganization of the biotin moiety on the surface. Interestingly, the lack of any binding switching ability in the pure biotin-4KC supports the hypothesis that there is a large steric hindrance around each oligopeptide, restricting conformational changes from taking place. As in the case of pure biotin-4KC, high ratios of biotin-4KC to TEGT limit the conformational change effects triggered by an electrical potential.

The switching efficiency increased significantly (Figure 2) as the proportion of biotin-4KC in the mixed monolayer decreases, reflecting the availability of local free volume for the conformational switching of the oligopeptides to occur on the gold surfaces. Interestingly, it is the fact that at low biotin-4KC:TEGT solution ratios, i.e., 1:100 and 1:500, the switching efficiency is lower than that at solution ratio of 1:40. This behaviour might be attributed to the formation of a more organized EG matrix on the mixed SAMs prepared from solutions containing low concentrations of the oligopeptide. The presence of a more packed EG matrix might restrict the oligopeptide mobility and conformational flexibility. Another point of note is that these suggested conformational changes are not only taking place at –0.4 V but also at +0.3 V, since the binding capacity of the latter was shown to be superior to that of open circuit (OC) conditions (i.e. no applied potential). From this observation, and the fact that the gap distance between the biotin moiety and the EG matrix is an important factor in determining the accessibility of biotin for neutravidin binding, as discussed above, we may infer that the conformational changes might induce: i) gap distance variations between the biotin and the EG matrix, and/or ii) changes in the orientation of the biotin that could make it more or less favourable for binding to neutravidin. We consider these two points in greater detail when we discuss below the results of the molecular dynamic simulations.

Next, we investigated the switching properties of the oligopeptide mixed SAM system as a function of the length of the oligolysine chain. A shorter (biotin-2KC) and a longer (biotin-6KC) oligopeptide than biotin-4KC were chosen as model systems (Scheme 6). Based on the switching studies performed on the biotin-4KC:TEGT mixed SAMs at different surface ratios, two oligopeptide:TEGT surface ratios were selected, namely 1:5 and 1:16. Apart from evaluating the switching abilities of the biotin-2KC and biotin-6KC, the use of an underperformed (1:5) and an optimum ratio (1:16) found for the biotin-4KC system allowed us to: i) evaluate the oligopeptides behavior in direct comparison and ii) unveil whether there is a relationship between the length of the oligolysine molecular switch and the molecular area that it requires for the switchable conformational changes to occur.

Mixed SAMs of different solution concentration ratios of Biotin-2KC or Biotin-6KC and TEGT, ranging from 1:10 to 1:2000, were prepared and analyzed by XPS (Figure S2). Following a similar procedure as described for the biotin-4KC, we were able to determine that ratios of 1:40 and 1:100 of biotin-2KC:TEGT mixed solution were required to provide biotin-2KC:TEGT surface ratios of 1:6 ± 1 and 1:16 ± 2, respectively. In a similar manner, the XPS studies allowed us to establish that ratios of 1:40 and 1:2000 of biotin-6KC:TEGT mixed solution are needed to achieve biotin-6KC:TEGT surface ratios of 1:7 ± 2 and 1:17 ± 2, respectively.

**Figure 3:**
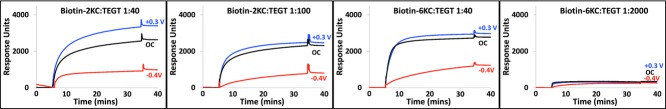
SPR sensorgram traces showing the binding of neutravidin (37 μg.mL^−1^) to the biotin-2KC:TEGT mixed SAMs (solution ratios of 1:40 and 1:100) and biotin-6KC:TEGT mixed SAMs (solution ratios of 1:40 and 1:2000) under open circuit conditions, an applied positive (+0.3 V) and negative (−0.4 V) potential.

The binding capacity and binding switching efficiency for these four mixed SAM surfaces are summarized in **Table** **2** and **Figure** **3**. A comparison of this experimental data with those obtained for the biotin-4KC:TEGT monolayers at same surface ratios, indicates that the binding capacity for the biotin-4KC and biotin-2KC systems are similar, while it is significantly impaired in the biotin-6KC:TEGT monolayers at a surface ratio of ≈1:16. We believe that such big difference in binding capacity is related to the unfavourable orientation of the biotin moiety in the biotin-6KC:TEGT mixed SAMs at low densities, caused by the long and flexible nature of the biotin-6KC.

**Table 2:** Binding capacity and switching efficiency as determined by SPR analysis for biotin-2KC:TEGT and biotin-6KC:TEGT at different surface ratios

	Ratio		Binding capacity (RU)	Switching efficiency (%)

	Solution	Surface		
**Biotin-2KC:TEGT**	1:40	1:6 ± 1	2634 ± 183	74 ± 4
	1:100	1:16 ± 2	2295 ± 87	67 ± 8
**Biotin-6KC:TEGT**	1:40	1:7 ± 2	2822 ± 129	71± 7
	1:2000	1:17 ± 2	229 ± 58	7 ± 4

In contrast to the low binding switching efficiency for the biotin-4KC:TEGT mixed SAM at a surface ratio of ≈1:5, the biotin-2KC:TEGT at the same surface ratio already demonstrated a great regulation of biomolecular interactions. This difference between the two oligopeptides can be rationalized by considering their molecular lengths (Scheme 6), and the need for a greater local free volume in the biotin-4KC:TEGT for the conformational switching of the oligopeptide to occur on the gold surface. Remarkably, the switching efficiency for the biotin-6KC:TEGT at a ≈1:5 surface ratio is also higher than for the biotin-4KC:TEGT, suggesting that for the longer oligopeptide (biotin-6KC) the conformation changes may induce intercrossing between oligopeptide chains leading to lower accessibility of biotin for neutravidin binding. In the case of the biotin-6KC:TEGT at a ≈1:16 surface ratio, the switching efficiency is significantly lowered relative to the two other systems, biotin-2KC:TEGT and biotin-4KC:TEGT, indicating that the length of the lysine switching unit exert great influence in the surface ratio that could provide maximum binding capacity and switching efficiency. These findings also suggest that a long switching unit can place constraints on the rearrangement of the biotin moiety on the surface, such that it can be available or not for neutravidin binding.

**Scheme 2:**
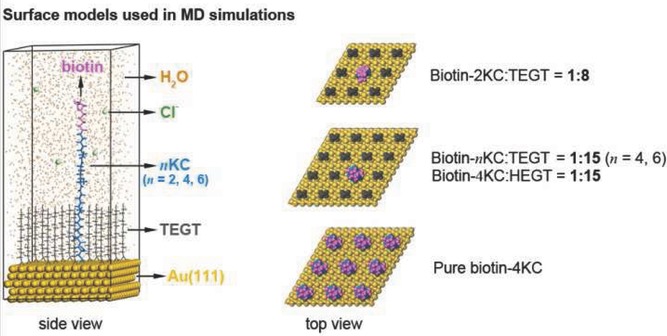
The surface models used in the MD simulations. The purple, blue and dark green parts of the biotin-*n*KC chain represent the biotin motif, lysine and cysteine residues, respectively. The orange dots, light green balls, yellow balls and short grey chains denote water molecules, chloride ions, gold atoms and TEGT, respectively.

In order to gain insight into the mechanism of the switching behavior, we performed molecular dynamics (MD) simulations. The performance of MD simulations depends mainly on the force field selected, and thus we tested three different force fields, namely cvff (consistent-valence force field), compass (condensed-phase optimized molecular potentials for atomistic simulation studies) and pcff (polymer consistent force field, see Supporting Information for details). The cvff force field performed best according to the test, and thus it was adopted in our simulations. The simulation models are shown in **Scheme** **2**. Two dimensional rhombic periodic boundary condition and slab models were applied throughout our simulations. Water mole­cules and chloride ions were adopted to simulate the PBS solution. Detailed model parameters are summarized in Table S1. External electric fields were applied to model the electric potentials used in the experiment. In order to consider the polarization caused by the electric field, density functional theory-derived partial charge was used. We carried out simulations for all the above-mentioned systems, including biotin-*n*KC:TEGT (*n* = 2, 4, 6), biotin-4KC:HEGT and pure biotin-4KC.

**Figure 4:**
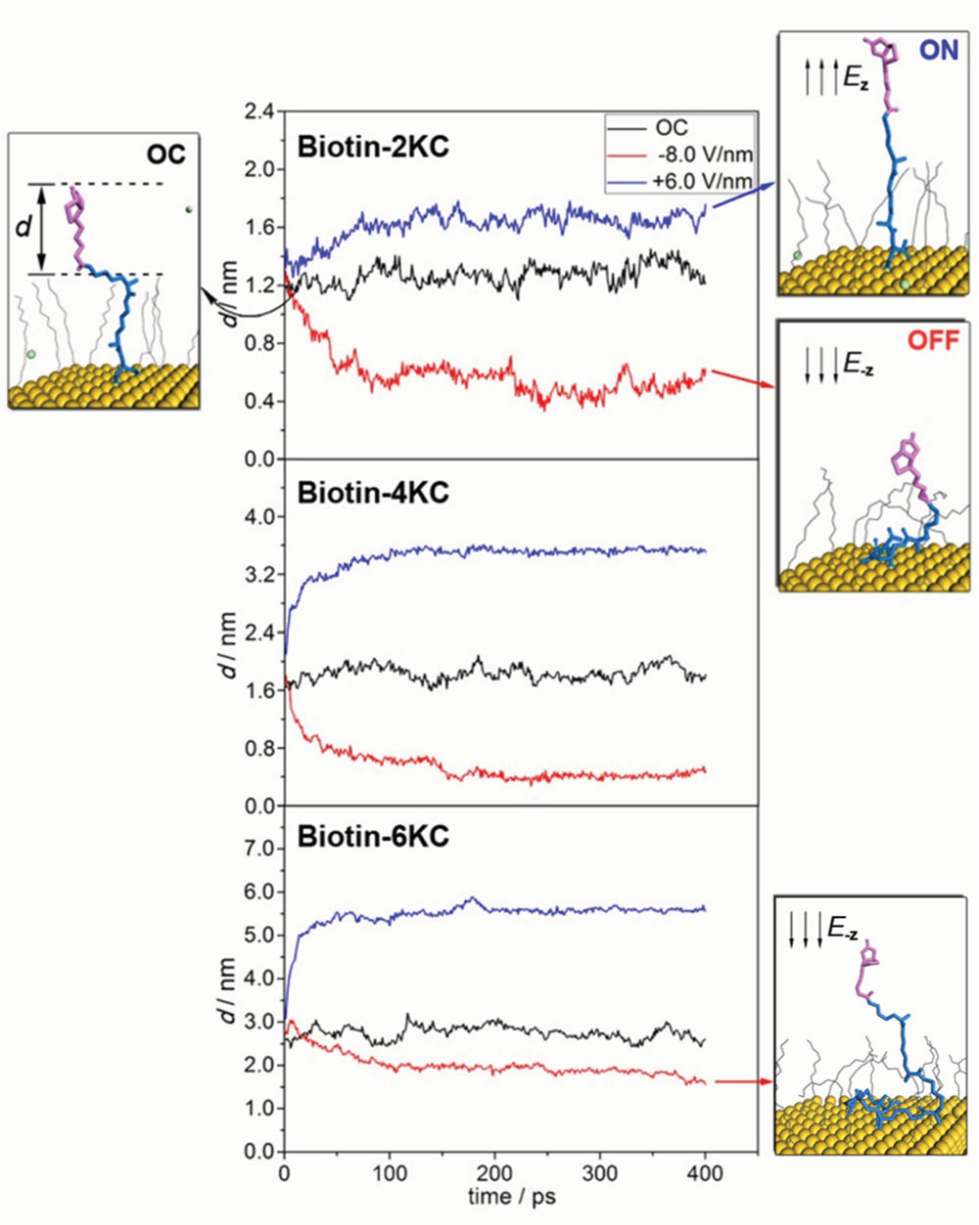
The conformational changes of biotin-2KC, biotin-4KC and biotin-6KC under different electric fields, along with the MD simulation snapshots. The black arrows show the directions of the applied electric fields. Water molecules and hydrogen atoms are omitted for clarity. *d* is defined as the gap distance variation between the biotin and TEGT matrix.

**Figure 5:**
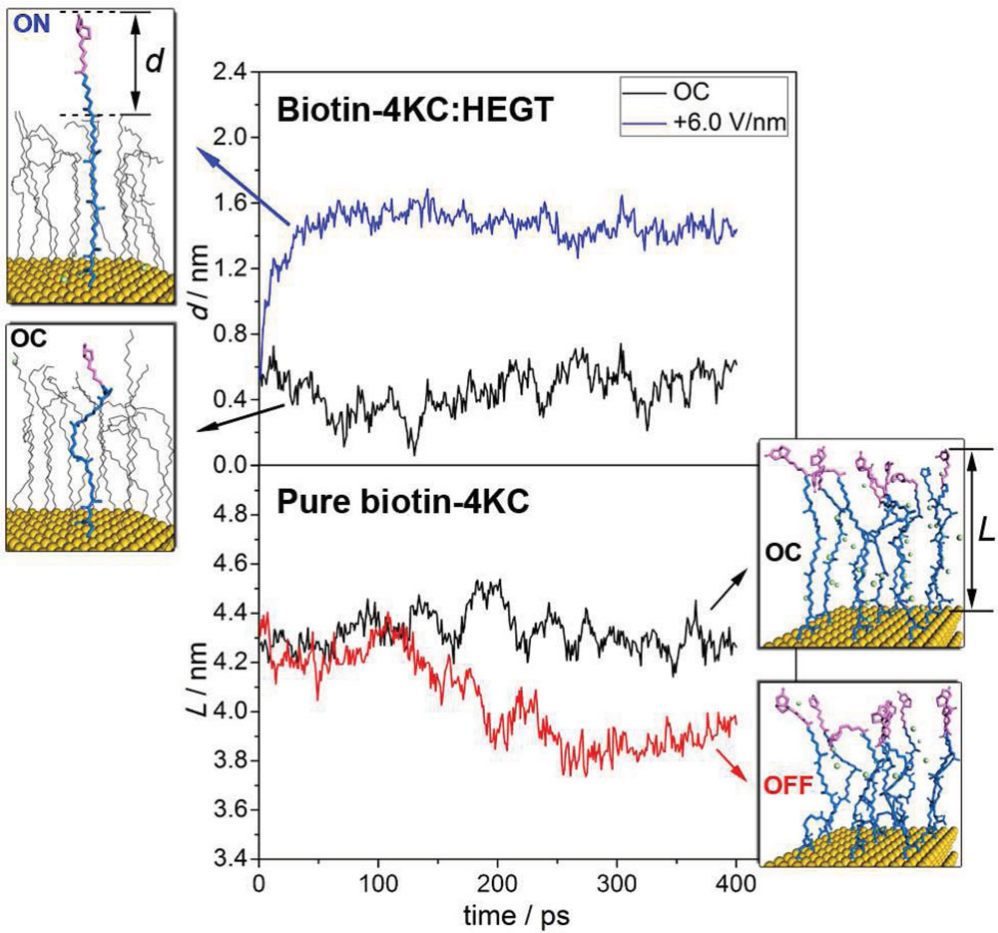
The conformational changes of biotin-4KC:HEGT (up) and pure biotin-4KC (down) under different electric fields, along with the MD simulation snapshots. *L* is defined as the gap distance variation between the biotin and gold surface.

The results are shown in **Figures** **4**–**5** and detailed MD simulation snapshots are listed in the Supporting Information. For the biotin-2KC:TEGT (surface ratio 1:8) and biotin-4KC (surface ratio 1:15), an evident switching behavior is observed. The oligopeptide chain extended fully and the biotin head (the purple part of the long central chain) was totally exposed when an electric field *E*_z_ was applied, corresponding to the “ON” state. In contrast, when *E*_-z_ was applied, the chain adopted a collapsed conformation and the biotin head was partially concealed by TEGT chains, thus showing no bioactivity (“OFF” state). The “OC” state took an intermediate conformation and the intercrossing between oligopeptide chains was more probable to occur, which would lower the chance of binding to neutravidin and result in moderate bioactivity.

These theoretical results are in fair agreement with the data obtained with the SPR experiments for the optimum ratios of biotin-2KC:TEGT and biotin-4KC:TEGT, which clearly revealed high binding at +0.3 V, reduced binding at −0.4 V, and intermediate binding in the OC conditions. The electrostatic interaction between the applied electric field and the positively charged lysine side chains, whose amino groups were fully protonated, is proposed to be the main driving force for these switchable conformational changes.

From Figure 4, we can see that in the 1:15 biotin-6KC:TEGT SAM the biotin-6KC chain was so long that even in the folded conformation the TEGTs could not conceal the biotin head. Another feature of note in Figure 4 is that, when an electric field *E*_z_ was applied to the biotin-6KC:TEGT mixed SAM, the biotin moiety is highly available for binding. This can be seen from the gap distance variation between the biotin and TEGT matrix (*d* in Figure 4), which is more than 1.5 nm. These results agree with the experimental data and confirm that the use of a long switching unit is not ideal for controlling ligand activity under an electrical stimulus.

In the case of biotin-4KC:HEGT (Figure 5), the ethylene glycol chains were long enough to cover partially the biotin in the OC condition (*d* < 0.5 nm). The biotin-4KC chain would extend to about 5.2 nm and reach neutravidin only when the *E*_z_ field was applied (*d* > 1.4 nm). This finding supports the interpretation that low neutravidin binding to the biotin-4KC:HEGT mixed SAM is an effect related with the biotin moieties standing to close to the ethylene glycol matrix, sterically shielding the biotin and making it inaccessible to the neutravidin. This hypothesis is also consistent with the conformational structures obtained for the biotin-2KC and biotin-4KC under *E*_-z_ and the low experimental binding obtained under a negative potential. The intercrossing between oligopeptide chains was more probable to occur in the OC condition, which would lower the chance of binding to neutravidin. Therefore, its bio-activity was lower than that in the ON condition. Thus, we now have a rationale for explaining how the switching mechanism present in an oligopeptide:TEGT mixed SAM can control binding capacity on the surface.

At this point, it is of interest to ask how oligopeptide density can affect the switching mechanism, and as a consequence, the binding switching efficiency. From Figure 5, it is noticeable that for the pure biotin-4KC SAM, the chains were closely packed on the surface and not sufficient space was left for the chains to collapse. The biotin heads were always exposed, leading to a persistent bioactivity, an observation that is consistent with the similar binding capacities at OC conditions, and applied negative and positive potential. We can infer from these findings that a basic criterion in the design of the switching surfaces is to provide sufficient freedom for conformational transitions of surface confined oligopeptide chains.

In our MD simulation, the electric field we adopted is an electrostatic field and no current was involved in the system. In the experiment, however, a circuit was formed and current was observed. The electrostatic field in our simulation was an approximation to the experimental electrical potential. The intensity of the adopted electric field was in the range of 10^0^ V/nm, which is commonly used in such kind of electro-switchable systems.

## 3. Conclusion

The switching mechanism on electrically switchable oligopeptide surfaces is based on conformational changes between collapsed (“OFF” state) and fully extended (“ON” state) oligopeptide structures. The principles behind the bioactivity OFF/ON switch can be understood in terms of the proximity of the biotin ligand to the EG matrix. When the oligopeptide is in its collapsed conformation, the biotin moiety draws closer to the EG matrix, hindering molecular recognition with the biotin in the binding pockets of neutravidin. In contrast, in the fully extended conformation, the biotin is largely free from steric hindrance effects and is able to efficiently bind to neutravidin. Our experimental and computational results strongly suggest that steric hindrances aroused from the neighboring surface-confined oligopeptide chains exert a great influence over the conformational behaviour of the oligopeptides, and as a consequence, over the binding switching efficiency. Our results also highlighted the relevance of the length of the switching unit to ensure maximum binding switching efficiency. Equipped with this kind of intimate understanding of the structure-switching property relationship, we are now better able to design and develop new dynamic surface materials for biomedical applications ranging in diversity from—but not limited to—in vitro model systems for fundamental cellular studies, all the way through to sophisticated drug delivery systems.

## Supporting Information

As a service to our authors and readers, this journal provides supporting information supplied by the authors. Such materials are peer reviewed and may be re-organized for online delivery, but are not copy-edited or typeset. Technical support issues arising from supporting information (other than missing files) should be addressed to the authors.
