# Supplementary material for: Modulation of Biointeractions by Electrically Switchable Oligopeptide Surfaces: Structural Requirements and Mechanism
Source: Adv Mater Interfaces. 2014 Jan 25;1(2):1300085. doi: 10.1002/admi.201300085 (PMC4358153; doi:10.1002/admi.201300085)
Supplement: Supplementary file 1 — Supplementary [file admi0001-1300085-sd1.pdf]

Copyright WILEY-VCH Verlag GmbH & Co. KGaA, 69469 Weinheim, Germany, 2014.

ADVANCED MATERIALS INTERFACES

Supporting Information

for *Advanced Materials Interfaces*, DOI: 10.1002/admi.201300085

Modulation of Biointeractions by Electrically Switchable Oligopeptide Surfaces: Structural Requirements and Mechanism

Chun L. Yeung, Xingyong Wang, Minhaj Lashkor, Eleonora Cantini, Frankie J. Rawson, Parvez Iqbal, Jon A. Preece, Jing Ma, and Paula M. Mendes**

Modulation of Biointeractions by Electrically Switchable Oligopeptide Surfaces: Structural Requirements and Mechanism

Chun L. Yeung,† Xingyong Y. Wang,§ Minhaj Lashkor,† Eleonora Cantini,† Frankie J. Rawson,† Parvez Iqbal,†,# Jon A. Preece,# Jing Ma,§,* Paula M. Mendes†,*

†School of Chemical Engineering and #School of Chemistry, University of Birmingham, Edgbaston, Birmingham, B15 2TT, UK.

§School of Chemistry and Chemical Engineering, Nanjing University, Nanjing 210093, P. R. China

Supporting information:

1. Chemicals and Materials

Commercially available chemicals and solvents were purchased from Aldrich Chemicals and Fisher Chemicals and were used as received. The oligopeptides biotin-2KC, biotin-4KC and biotin-6KC were synthetised by Peptide Protein Research Ltd. (Wickham, UK) to > 95% purity and verified by HPLC and mass spectrometry. Neutravidin was purchased from Invitrogen. Triethylene glycol thiol was synthesized as previously described.[1] Phosphate buffered saline (PBS) solution was prepared from a 10× concentrate PBS solution (1.37 M sodium chloride, 0.027 M potassium chloride, and 0.119 M phosphate buffer) from Fisher BioReagents. For the XPS analysis, polycrystalline gold substrates were purchased from George Albert PVD., Germany and consisted of a 50 nm gold layer deposited onto a glass covered with a thin layer of chromium. SPR gold chips were purchased from Reichert Technologies, US.

2. SAM Preparation

The gold substrates were cleaned by immersion in piranha solution (3:1, H_2SO_4 : 30% H_2O_2) at room temperature for 10 min, rinsing with Ultra High Pure (UHP) H_2O and then HPLC grade EtOH thoroughly for 1 min. (*Caution: Piranha solution reacts violently with all organic compounds and should be handled with care*). For the preparation of the pure biotin-4KC SAMs, the clean gold substrates were immersed for 12 h in ethanolic 0.1 mM solution of biotin-4KC containing 3% (v/v)

$N(CH_2CH_3)_3$. For the preparation of the mixed oligopeptide:TEGT SAMs, solutions of the oligopeptide (0.1 mM) and TEGT (0.1 mM) were prepared in HPLC EtOH containing 3% (v/v) $N(CH_2CH_3)_3$, and mixed at different volume ratios. Subsequently, the clean gold substrates were immersed in the mixed solution for 12 h to form the mixed SAMs on the gold surfaces. The substrates were rinsed with HPLC EtOH, an ethanolic solution containing 10% (v/v) CH_3COOH, and UHP H_2O and dried under a stream of N_2. Note that the mixed SAMs were deposited in the presence of $N(CH_2CH_3)_3$ to prevent the formation of hydrogen bonds between the NH_2 functional groups of the bound thiolate peptide on Au surface and that of free thiol peptide in the bulk solution.[2]

3. Electrochemical Surface Plasmon Resonance (SPR)

SPR switching experiments were performed with a Reichert SR7000DC Dual Channel Spectrometer (Buffalo, NY, USA) at 25°C using a three-electrode electrochemical cell and a Gamry PCI4/G300 potentiostat. The SAMs prepared on Reichert Au sensor chips served as the working electrode, the counter electrode was a Pt wire, and a standard calomel electrode (SCE) was used as the reference electrode. Prior to the neutravidin binding studies, the sensor chips were equilibrated with degassed PBS, followed by application of either + 0.3 V, − 0.4 V or open circuit conditions for 10 min while passing degassed PBS through the electrochemical cell at a flow rate of 100 $\mu L\ min^{-1}$. While still applying a potential, neutravidin (500 µ L, 37 $\mu g\ mL^{-1}$), was injected over the sensor chip surface for 10 s at 1500 $\mu L\ min^{-1}$ and then 30 min at 8 $\mu L\ min^{-1}$ (the decrease in flow rate from 1500 to 8 $\mu L\ min^{-1}$ ensures that sufficient exposure time is provided for binding to occur between the biotin on the surface and neutravidin in solution). In order to remove any unbound neutravidin, the sensor chips were washed with degassed PBS for 10 s at a flow rate of 1500 $\mu L\ min^{-1}$, followed by 20 min at a flow rate of 100 $\mu L\ min^{-1}$ while still applying a potential to the chips. The averages and standard errors reported were determined from at least three different SPR measurements.

4. X-ray photoelectron spectroscopy (XPS)

XPS spectra were obtained on the VG Escalab 250 instrument based at University of Leeds EPSRC Nanoscience and Nanotechnology Facility, UK. XPS experiments were carried out using a monochromatic Al K α X-ray source (1486.7 eV) and a take-off angle of 15°. High-resolution scans of N (1s) and S (2p) were recorded using a pass energy of 150 eV at a step size of 0.05 eV. Fitting of XPS peaks was performed using the Avantage V 2.2 processing software. Sensitivity factors used in this

study were: N (1s), 1.73; S (2p), 2.08; Au (4f 7/2), 9.58; Au (4f 5/2), 7.54. The averages and standard errors reported were determined from at least four different XPS measurements.

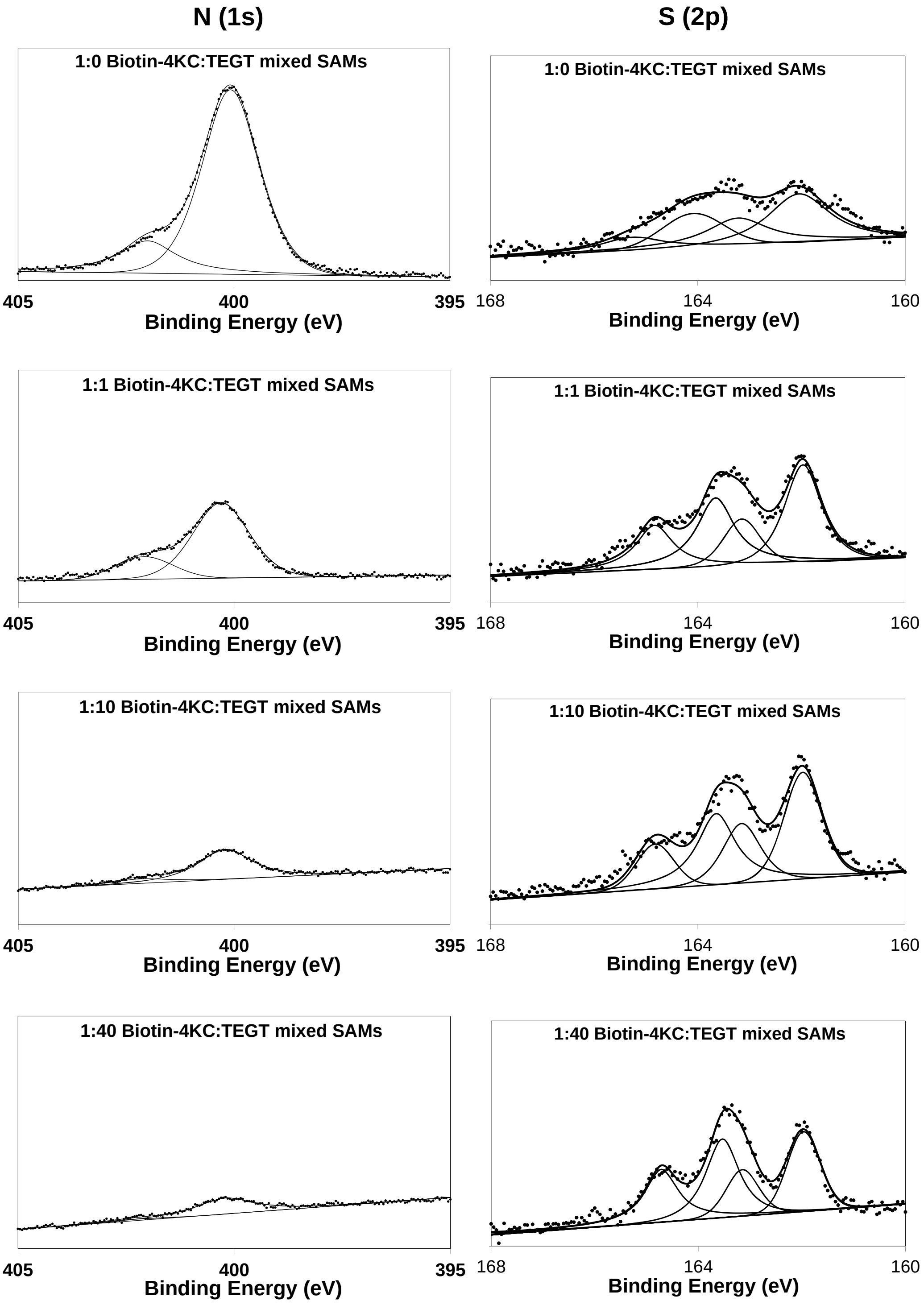

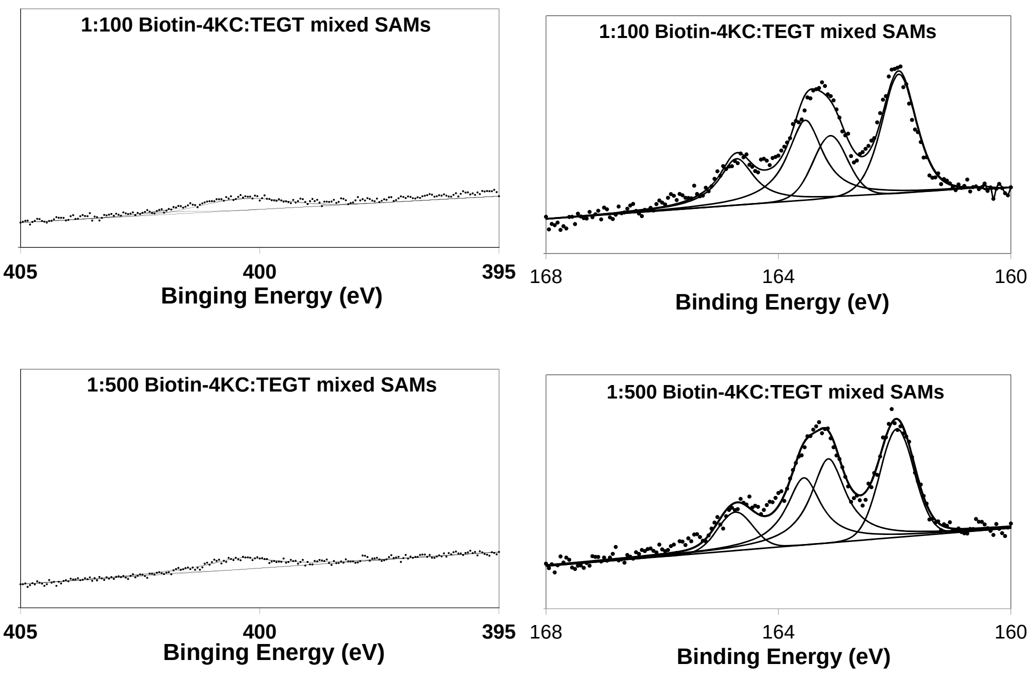

Figure S1. XPS spectra of the N (1s) and S (2p) peak regions of mixed SAMs of different solution ratios of biotin-4KC and TEGT – 1:0, 1:1, 1:10, 1:40, 1:100 and 1:500.

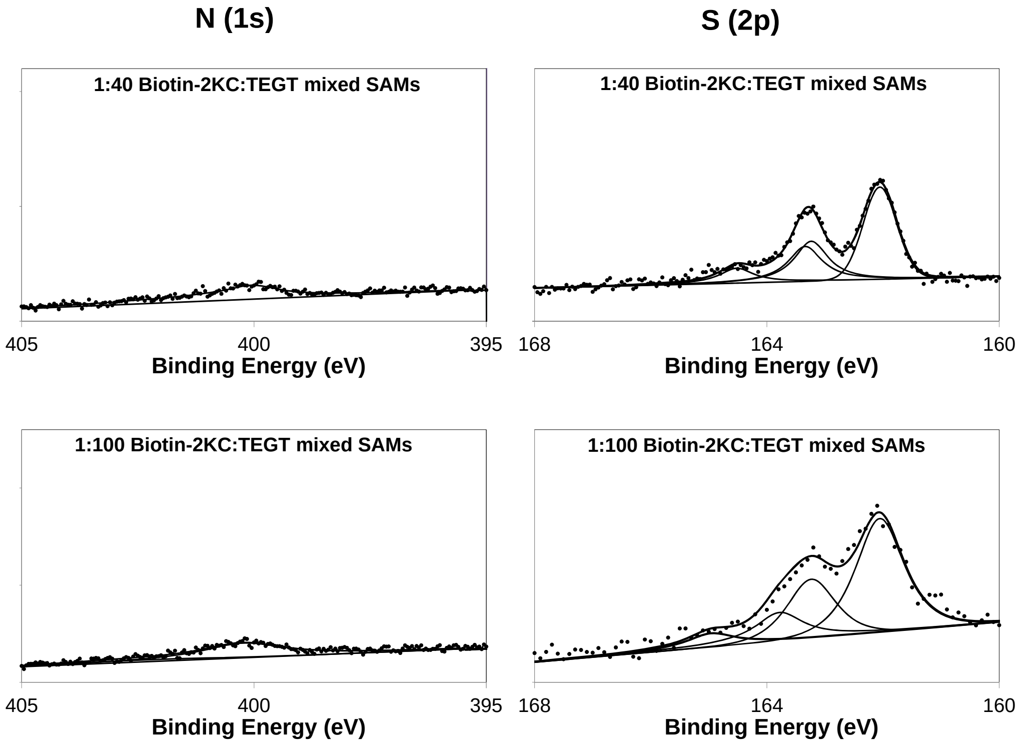

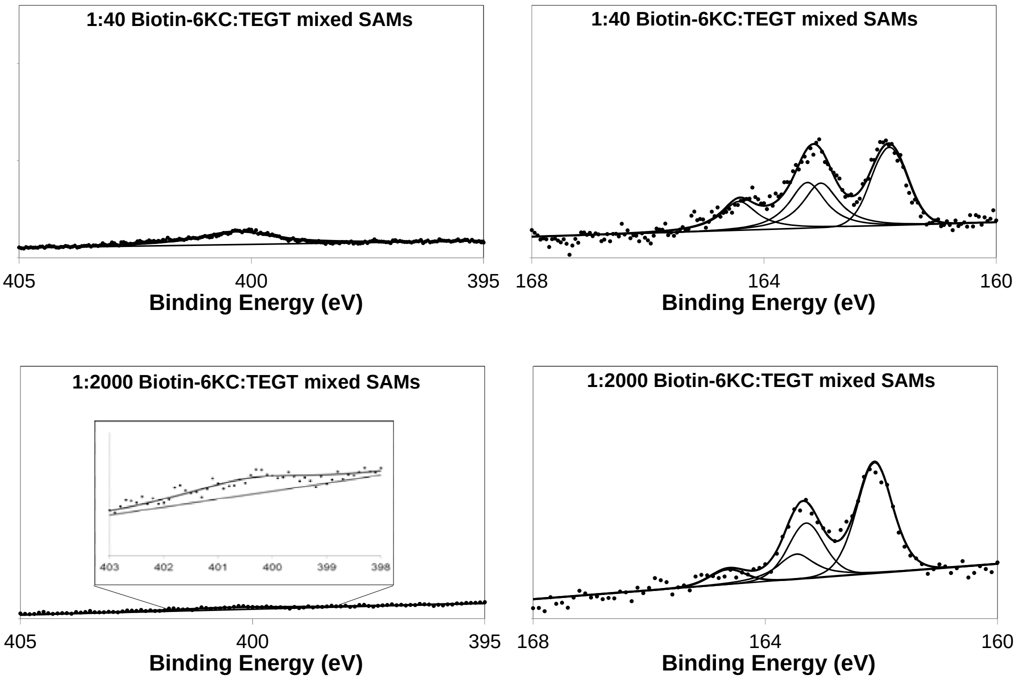

Figure S2. XPS Spectra of the N (1s) and S (2p) peak regions of biotin-2KC:TEGT mixed SAMs at solution ratios of 1:40 and 1:100, and biotin-6KC:TEGT mixed SAMs at solution ratios of 1:40 and 1:2000.

5. Force field test

Since the conformational switching of biotin-*n*KC chains mainly results from the rotation of the C-C bonds, the energy scan for biotin-4KC molecule with different C1-C2-C3-C4 dihedrals (θ, Fig. S3) was carried out by both force field methods and density functional theory (DFT) calculations with the B3LYP functional and 6-31G(d) basis set. Three kinds of force fields, cvff, compass and pcff were tested. The result is shown in Figure S3. The cvff force field shows the best performance. Although it overestimates the energies compared to the DFT result, it displays the right shape of the energy curve. In contrast, both compass and pcff force fields result in a significant deviation from the DFT result. So the cvff force field was adopted throughout our simulations.

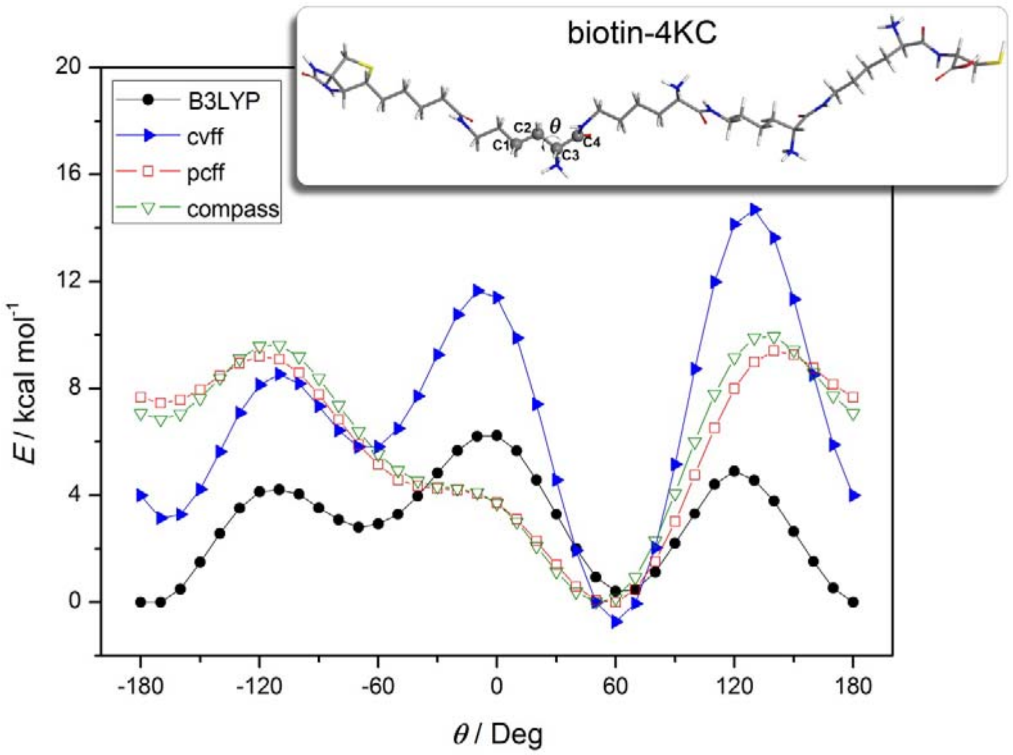

Figure S3. The energy scanning for biotin-4KC molecule with different C1-C2-C3-C4 dihedrals, θ, obtained by both force field methods and DFT calculations.

Table S1. Parameters for the surface models used in the simulations.

Surface chains	Solvent molecules (H_2O)	Ions (Cl^-)	Cell parameters (Å^3)
Biotin-2KC/8(TEGT)	957	2	25.95 × 25.95 × 65.42
Biotin-4KC/15(TEGT)	2115	4	34.60 × 34.60 × 77.42
Biotin-6KC/15(TEGT)	2728	6	34.60 × 34.60 × 95.42
Biotin-4KC/15(HEGT)	1974	4	34.60 × 34.60 × 77.42
9(Biotin-4KC)	1982	36	34.60 × 34.60 × 77.42

6. Computational details

Five layers of gold atoms cut from the Au(111) surface were adopted to model the gold substrates used in the experiment and they were fixed during the simulations. All MD simulations were performed in the canonical (NVT) ensemble using the cvff force field. The temperature was set to 298 K by using the Andersen thermostat.[3] The equations of the motion were integrated using the velocity Verlet algorithm[4] with the time step of 1fs. The atomic charges for the biotin-*n*KC molecules were updated every 100ps by DFT calculations, at the M06-2X/6-31G(d,p) level of theory. The Discover module in the Materials Studio package[5] was employed to run all the MD simulations. All DFT calculations were carried out with the Gaussian 09 program package.[6]

7. MD simulation snapshots for biotin-4KC and biotin-6KC under different electric fields

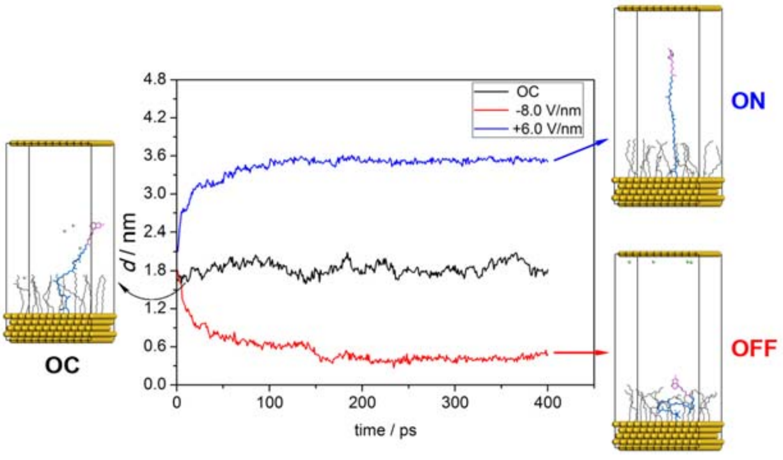

Figure S4. The conformational change of bioin-4KC under different electric fields, along with the MD simulation snapshots.

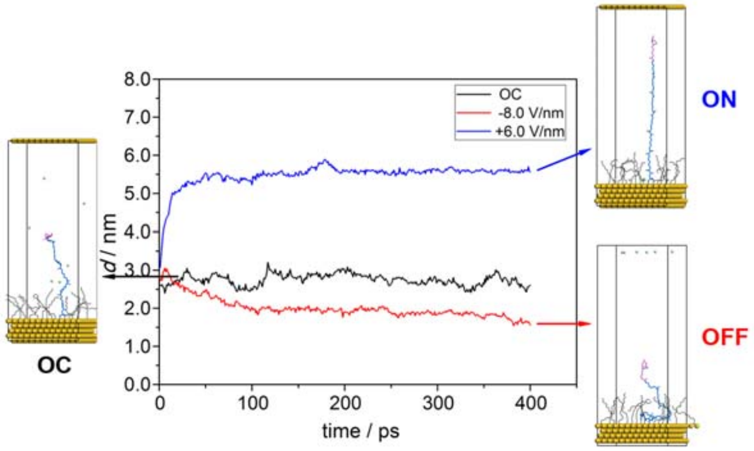

Figure S5. The conformational change of biotin-6KC under different electric fields, along with the MD simulation snapshots.

References

(1) Yeung, C. L.; Iqbal, P.; Allan, M.; Lashkor, M.; Preece, J. A.; Mendes, P. M. *Adv. Funct. Mater.* **2010,** *20*, 2657–2663.

(2) H. Wang, S. F. Chen, L. Y. Li, S. Y. Jiang, *Langmuir* **2005**, *21*, 2633.

(3) Andersen, H. C. *J. Chem. Phys.* **1980**, *72*, 2384-2393.

(4) Allen, M. P.; Tildesley, D. J. *Computational Simulation of Liquids*; Oxford University Press: New York, USA, 1987.

(5) Materials Studio, version 4.0, Accelrys Inc., San Diego, 2006.

(6) Gaussian 09 (Revision B.01), Frisch, M. J. *et al.* Gaussian, Inc., Wallingford CT, **2009**.
